# Droplet Characterization and Penetration of an Ultra-Low Volume Mosquito Adulticide Spray Targeting the Asian Tiger Mosquito, *Aedes albopictus*, within Urban and Suburban Environments of Northeastern USA

**DOI:** 10.1371/journal.pone.0152069

**Published:** 2016-04-26

**Authors:** Ary Faraji, Isik Unlu, Taryn Crepeau, Sean Healy, Scott Crans, Griffith Lizarraga, Dina Fonseca, Randy Gaugler

**Affiliations:** 1 Center for Vector Biology, Department of Entomology, Rutgers University, New Brunswick, New Jersey, United States of America; 2 Salt Lake City Mosquito Abatement District, Salt Lake City, Utah, United States of America; 3 Monmouth County Mosquito Extermination Commission, Eatontown, New Jersey, United States of America; 4 Department of Entomology, Louisiana State University, Baton Rouge, Louisiana, United States of America; 5 Department of Environmental Sciences, Clarke Mosquito Control, Roselle, Illinois, United States of America; Cary Institute of Ecosystem Studies, UNITED STATES

## Abstract

Adult control of *Aedes albopictus* via ultra-low volume is difficult because this species occurs primarily in peridomestic habitats where obstacles such as buildings and vegetation can disrupt spray plumes and droplet dispersion. We determined droplet penetration and characterization of a pyrethroid adulticide applied from the ground at mid (46.77 ml/ha) and maximum (93.53 ml/ha) label rates within cryptic habitats of urban and suburban environments. Droplets were collected from all habitats, with no significant differences detected between locations within the same application rate or collection method. No differences were detected in droplet densities (drops per mm^2^) between rates within urban environments, but more droplets were collected in urban (149.93 ± 11.07 SE) than suburban sites (114.37 ± 11.32) at the maximum label rate (*P* = 0.003). The excellent penetration of aerosols into cryptic habitats of an urban site was likely due to the shorter spray paths afforded by our network of roads and alleys. Mid label rates displayed similar droplet density values as maximum label rates in urban areas, indicating that lower rates may be used effectively to reduce costs, lessen non-target effects, and increase environmental stewardship. Advances in formulations and technology are driving changes in adulticide applications, leading to use of the minimum effective dose for maximum efficacy, precision, and accountability.

## Introduction

With growing globalization and commerce, mosquito invasions are increasing worldwide.[1–3] However, concerns for the environment and society, beckon the need to lessen the environmental impact of insecticides used to control insect vectors. Nonetheless chemical control, particularly adulticides applied as ultra-low volume (ULV) cold aerosol space sprays, remain as the only effective means of reducing transmission risk to humans during arboviral disease epidemics or when vector population densities are high.[4]

This is particularly important for the Asian tiger mosquito, *Aedes albopictus* (Skuse), which is among the most invasive and aggressive disease vectors in the world.[5] The range of this species is currently still expanding, particularly into highly dense human population centers in temperate urban and suburban areas, raising the public health threat of emerging and re-emerging diseases such as chikungunya and dengue.[1,6] This may be particularly imminent in the case of CHIKV, as the virus is explosively spreading in the Caribbean region of the western hemisphere for the first time.[7] The immatures of this species exploit artificial containers found in human peridomestic environments and the day-biting adults concentrate in parks and tree-lined backyards, a staple of most American cities.[8,9] Urban mosquitoes are difficult to control because access to infested private properties is limited and the larval habitats are ubiquitous within the urban landscape. Consequently, area-wide ULV insecticide sprays may be the only effective method to protect urban areas from *Ae*. *albopictus*.[10]

The aim of an ULV application is to deliver the most efficacious droplet size using the least amount of insecticide that will control the target mosquitoes.[11] ULV adulticide applications are conducted in the evening or early morning when a thermal inversion has occurred and light winds are present to aide in droplet carry. ULV applications have often been ineffective in controlling diurnally active urban mosquitoes, such as *Aedes aegypti* (L.) and *Ae*. *albopictus*. Previous researchers have hypothesized that this lack of control may be a result of resting behavior, allowing gravid or engorged females to remain sequestered during nighttime ULV applications in cryptic habitats that are sheltered from the insecticide plume.[12–16] Crucial information is lacking regarding penetration and density of aerosolized spray droplets within urban and suburban environments where buildings and vegetation can disrupt the movement of the spray plume. Few studies have been conducted to evaluate aerosolized droplet dynamics and characterization during real world spray applications. There is a conflicting increase in the public awareness and environmental concerns regarding insecticides versus the imminent risk to public health of an *Ae*. *albopictus*-driven arboviral epidemic. Consequently vector control officials must be prepared in all aspects of their integrated mosquito management (IMM) approaches to intervene with the most effective products and application strategies. A critical need exists for novel methods of insecticide application or new formulations to achieve successful control while maintaining environmental stewardship and accountability.

We evaluated and characterized the penetration and droplet dynamics of an ULV cold aerosol application of a novel adulticide at mid (46.77 ml/ha) and maximum (93.53 ml/ha) label rates within urban and suburban residential communities in temperate North America. Specifically, we were interested in determining whether the spray droplets could penetrate vegetation and structural barriers to reach cryptic resting locations where diurnally active *Ae*. *albopictus* may be resting during a nocturnal application. We also compared the deposition efficacy of two different rotating impactors used to measure droplet density (per unit area) and distribution. Lastly, we compared two different techniques (digital image analysis versus traditional manual microscope readings) used to quantify droplets collected on rotating impactors.

## Materials and Methods

No specific permits were required for the described field studies, which were developed with homeowners assent by professional county mosquito control personnel. These studies did not involve endangered or protected species.

### Experimental sites

#### Urban site selection

**Fig 1:**
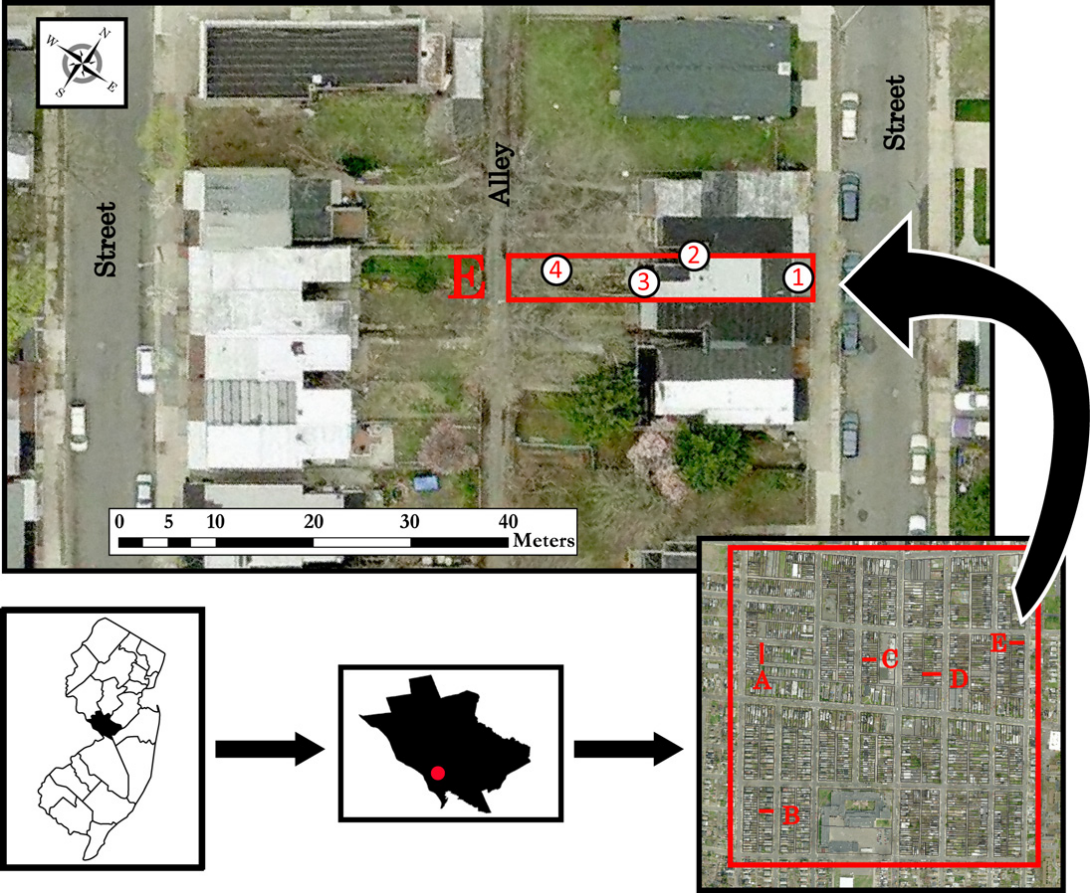
Droplet sampling locations in urban Mercer County, New Jersey, USA. Five parcels were selected within a 48.6 ha plot (A through E) and four stations were sampled within each parcel (1 = Front, 2 = Alcove, 3 = Porch, 4 = Backyard). Figure was created in ArcGIS 10.2 (ESRI, Redlands, CA).

A highly urbanized residential field site was chosen in Mercer County, New Jersey, USA (40° 13’ N, 74° 44’ W) as part of an area-wide management of the Asian tiger mosquito. Detailed descriptions about site selection and demographics have been published previously.[10,17,18] We used ArcGIS (10.2, ESRI, Redlands, CA) to display the experimental field site (urban Mercer) located in Trenton, New Jersey, in an area of low income housing (human population density of 4,286.5 /km^2^) and consisting of 48.6 ha, including 1,251 parcels with an average parcel size of 199.5 ± 18.3 m^2^ (Fig 1). Parcels correspond to a structure or house with surrounding yard, and are most often built as adjoining row homes or duplexes. Most parcels contain a sheltered alcove area between two homes, where small shrubs and trash proliferate, affording a shaded and humid area for a cryptic resting place (S1 Fig). Our field site consists of roughly 26 residential blocks, each containing a residential street on all four sides, and divided by a drivable alley between parallel parcels (Fig 1). During ULV adulticide applications, both streets and alleys were driven to maximize insecticide dispersal. Within our 48.6 ha urban site, we selected five random parcels (designated as A, B, C, D, E within Fig 1) for use during droplet sampling. Each parcel was either part of a row home or a duplex, containing an alcove area of interest (S1 Fig). Within each parcel, we selected four stations to be used during sampling and assigned them as Front, Alcove, Porch, and Backyard (Fig 1). The Front and Backyard stations were closest to the line of application, since the truck-mounted sprayer drove both the street and alley. However, the Backyard station was mostly surrounded by vegetation and chain link fencing which enclosed the yard. The Porch station was within the yard, closest to the back of the home, and the Alcove station was the most sheltered location, being completely enclosed by the front of the home and only accessible from the backyard (Fig 1, S1 Fig).

#### Suburban site selection

**Fig 2:**
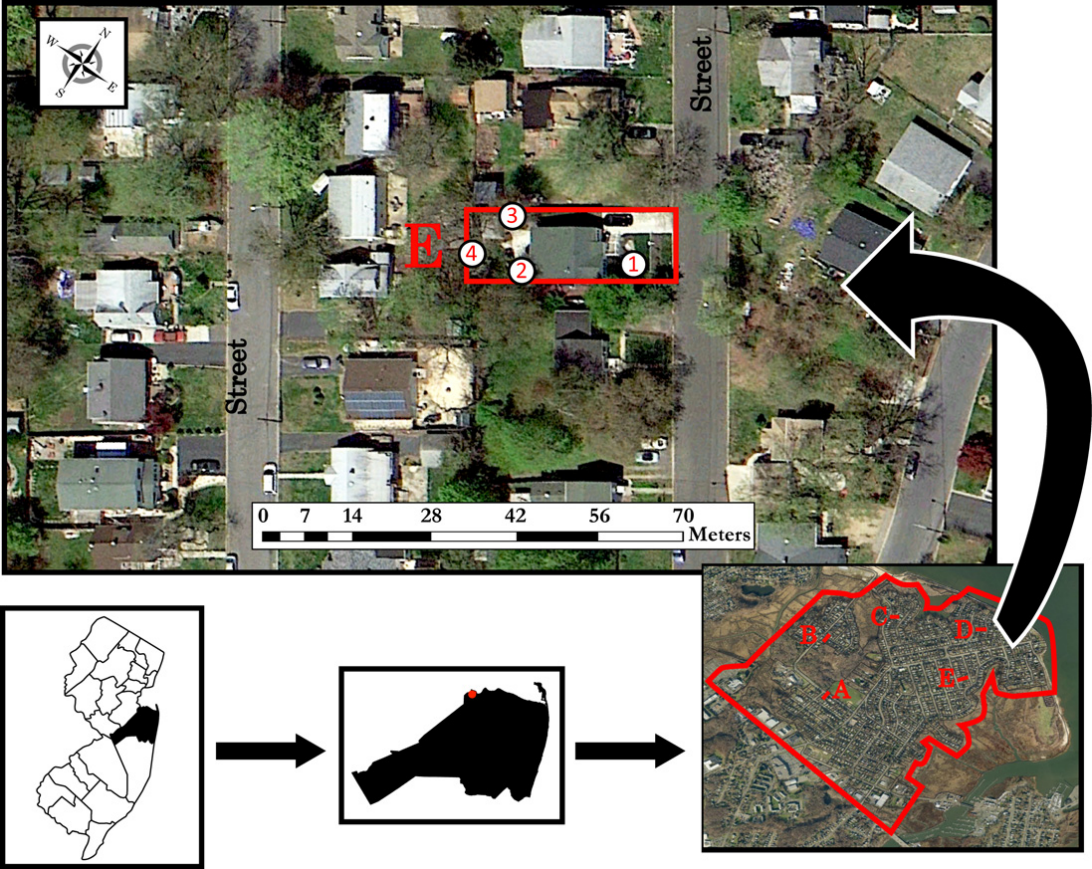
Droplet sampling locations in suburban Monmouth County, New Jersey, USA. Five parcels were selected within a 156.1 ha plot (A through E) and four stations were sampled within each parcel (1 = Front, 2 = Porch, 3 = Mid Yard, 4 = Backyard). Figure was created in ArcGIS 10.2 (ESRI, Redlands, CA).

A suburban residential field site was chosen in Monmouth County, New Jersey, USA (40° 26’ N, 74° 13’ W).[17,18] The field site (suburban Monmouth) is located within Cliffwood Beach in the boroughs of the Raritan Bayshore (human population density of 1,907.4 /km^2^) and consists of 156.1 ha, including 1,247 parcels with an average parcel size of 571.1 ± 31.2 m^2^ (Fig 2). Parcels in this field site are single housing structures primarily composed of middle income coastal suburban homes which are often interspersed with forest and green space remnants. This field site consists of roughly 60 residential blocks, many of which do not include a residential street on all four sides, and none of which are divided by a drivable alley (Fig 2). During ULV adulticide applications, only the streets were driven to disperse the aerosolized insecticide. Within our suburban site, we also selected five random parcels (designated as A, B, C, D, E within Fig 2) for droplet sampling. Within each parcel, we selected four stations and assigned them as Front, Porch, Middle Yard, and Backyard (Fig 2). The Front station was closest to the line of application and the Backyard furthest, since the truck-mounted sprayer could only be applied from the street.

### Ultra-low volume insecticide application

#### Spray boom set-up and calibrations

A Cougar^®^ (Clarke Mosquito Control, Roselle, IL) cold aerosol ULV generator was used for applications. The sprayer was fitted with a SmartFlow^®^ (Clarke Mosquito Control) system used in tandem with ground speed of the vehicle to ensure appropriate flow of insecticide and accurate reporting and tracking of amount of chemical used along with distance and area sprayed. The sprayer was mounted on a flatbed truck at a height of 1.8 m, and the spray boom was angled 45.5° backwards.

Droplet size and distribution are two of the most important factors affecting the success of an ULV application.[19] Droplet size measurements were obtained for the sprayer prior to applications using a DC-III™ portable droplet measurement system (KLD Laboratories, Huntington Station, NY). For adult mosquito control, a droplet size range of 5 to 25 μm is most efficient, because this size is most likely to stay adrift and impinge on a mosquito and deliver a toxic dose.[20] Droplet measurements are often provided as a mass median diameter or volume mean diameter (VMD). The VMD is also provided as DV_0.5_ to represent where 50% of the spray volume or mass is contained in droplets smaller than this value. Values for a DV_0.1_ and a DV_0.9_ are also often provided to describe 10% and 90% of the cloud volume, respectively. Additionally, adulticide labels require that equipment adhere to required VMD values. We conducted two readings using the DC-III during our calibration of the Cougar ULV sprayer and acquired a DV_0.1_ value of 2.88 μm, a VMD (DV_0.5_) value of 15.18 μm, and a DV_0.9_ value of 30.82 μm. A total of 4,015 drops were counted, with only 6 droplets above 32 μm in size, and none above 48 μm.

#### Insecticide and ULV application

We used a novel adulticide, DUET™ Dual-action Adulticide (Clarke Mosquito Control), which causes a benign agitation that potentially flushes mosquitoes from resting places and increasing contact with airborne droplets.[21,22] DUET adulticide combines the pyrethroids sumithrin (5%, 43.02 g Active Ingredient per L) and prallethrin (1%, 8.59 g AI per L) with the synergist piperonyl butoxide (5%, 43.02 g AI per L). Prallethrin induces an excitatory response at sublethal concentrations and exposes mosquitoes to lethal doses of airborne sumithrin and piperonyl butoxide.[21,23] This adulticide may have advantages against not only resting gravid or engorged mosquitoes, but also diurnal mosquitoes such as *Ae*. *albopictus* which may be inactive during nighttime ULV applications.

The pesticide label for DUET requires ground-based spray equipment to be adjusted to deliver aerosolized droplets within a VMD of 8 to 30 μm (DV_0.5_ < 30 μm) and a DV_0.9_ value of less than 50 μm. DUET was applied at a flow rate of 136.04 ml/min. Applications were conducted at the mid and the maximum label rates recommended on the DUET label. The mid label rate for a ground ULV application of DUET is 46.77 ml/ha, resulting in 0.40 g AI per ha of prallethrin, 2.02 g AI per ha of sumithrin, and 2.02 g AI per ha of piperonyl butoxide. The maximum allowable label rate is 93.53 ml/ha, which delivers 0.81 g AI per ha of prallethrin, 4.04 g AI per ha of sumithrin, and 4.04 g AI per ha of piperonyl butoxide. In urban Mercer, we conducted an application at the mid label rate and a second application at the maximum label rate, while in suburban Monmouth, we made a single application at the maximum label rate. In order to limit corruption of collection slides with other airborne pollutants (e.g., sap, dust, dew, fuel residue, etc.) the fluorescent tracer dye Uvitex^®^ OB (Tinopal^®^ OB, Ciba Corporation, Newport, DE) was mixed with the pesticide at a 0.125% weight to volume ratio, or 1.32 g/L. This dye does not alter pesticide formulation properties, droplet spectrum, or movement of pesticide droplets in the environment.[24]

Because of the complexity and logistics involved in an area-wide metropolitan application, treatments were made at night (2:30 to 5:00 a.m.) when human activity was minimal. A single vehicle was driven at an average speed of 16.1 km/h and spray routes were designed to follow roads and alleys to maximize coverage. Adulticide labels stipulate

### Aerosol sample collection

#### Rotating impactors

**Fig 3:**
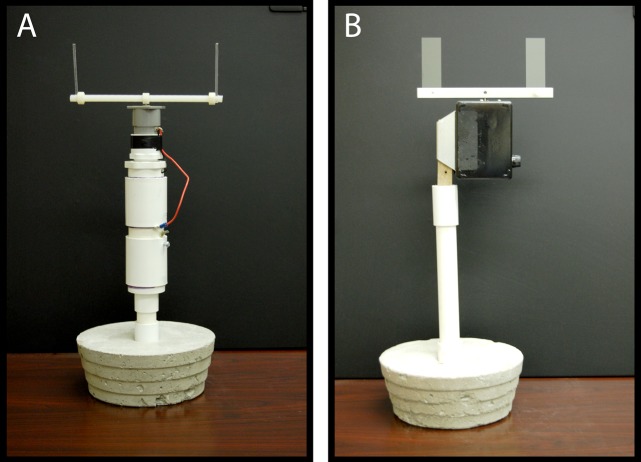
Rotating impactors used for droplet sampling of adulticidal spray plumes. A) Florida Latham Bonds (FLB) rotary-type impactor with 3 mm rods. B) Hock rotary impactor with 25 mm microscope slides.

Rotating impactors are devices for collecting and measuring droplet density, size, and distribution. The standard impactor used in mosquito control has been the Hock™ impactor (J.W. Hock, Gainesville, FL) which uses 25 mm wide Teflon-coated microscope slides at a rotational velocity of 3.6 m/sec (Fig 3). However, this type of impactor is inefficient at collecting the smaller size droplets produced in adulticide applications.[25,26] A more robust impactor has been developed, the Florida Latham Bonds (FLB) impactor[27], which uses 3 mm Teflon-coated acrylic rods (slides) rotating at 5.6 m/sec (Fig 3). In laboratory comparative assays, the FLB sampler had a higher droplet size distribution when compared to the Hock sampler across three wind speeds (1, 1.8 and 3.5 m/sec).[25] In short, FLB impactors collect much higher densities of smaller aerosolized droplets under laboratory conditions. We deployed 20 Hock and 20 FLB impactors[27] for our field evaluations. Each impactor uses two slides, and both impactors were placed at each station at ground level, resulting in 80 slides for measurement after each application (5 parcels x 4 stations x 2 impactors x 2 slides each).

#### Droplet size and density determination

Slides were collected 1 h post-application and immediately placed individually inside enclosed Styrofoam coolers to limit evaporation of impinged drops. All slides were transported to the laboratory and read within 12–48 h post-application. The DropVison^®^-Fluorescence (Leading Edge Associates, Waynesville, NC) program is a measuring system that digitally reads slides through proprietary image analysis. The software eliminates background particles, coalesced droplets, or non-qualified drops, and only recognizes droplets that contain the dye tracer. Slides were read by two experienced staff members under 100x microscopy. Approximately 1,000 drops from each FLB station (500 per slide), and 400 drops from each Hock station (200 per slide) were counted.

To compare data obtained through this digital approach to more standard manual analysis of droplets, we only used Hock impactors. Hock impactors utilize traditional microscope slides and are often used by mosquito control personnel for spray characteristic investigations. However, manual readings of slides is labor intensive and may average >30 min for analysis of 200 drops on a single slide. We analyzed all Hock slides from urban Mercer applications conducted at the mid label and maximum label rates.

### Meteorological data collection

In Mercer, meteorological data during testing was recorded for wind speed, direction, humidity, and temperature at 1 m and 10 m heights for thermal inversion observation. A Vantage Pro2™ (Davis Instruments, Hayward, CA) portable weather station was utilized during each application and set up within the treatment site 14 h prior to application and maintained until 8 h post application. A permanent weather station (KTTN) located <1 km from application site in Trenton, was used for additional meteorological data. In suburban Monmouth, meteorological data was acquired from a permanent weather station (KNJKEYPO2) located within our application site at Keyport.

### Statistical analysis

We determined droplet penetration, density, and size (DV_0.5_) by analyzing ~1,000 drops from each FLB impactor (~500 drops per slide) and ~500 drops from each Hock impactor (~250 per slide). Droplet characteristics were combined by location (Front, Alcove, Porch, Backyard) for each of the five parcels sampled to determine the mean value for each application rate and county. Differences between means were examined using two-way analysis of variance (ANOVA) with an accepted level of significance for all comparisons of *P* < 0.05 (SPSS version 18, IBM Corp, Armonk, NY).

## Results

### Aerosolized spray droplet penetration and characterization

#### Urban Mercer County

*FLB rotating impactors*. During mid label rate applications, we analyzed over 23,000 droplets from all slides, with a mean value of 1,156 drops per station and 578.1 drops per slide (S2 Fig). During maximum label rate applications, we analyzed over 19,600 droplets from all slides, with a mean value of 982.5 drops per station and 491.3 drops per slide (S3 Fig). We collected droplets consistently from all four stations (Front, Alcove, Porch, Backyard) with no significant differences in droplet density observed by rate (*F* = 2.07; df = 1; *P* = 0.160), location (*F* = 0.42; df = 3; *P* = 0.74), or rate within location (*F* = 0.05; df = 3; *P* = 0.99) (Table 1). Although no differences were observed in VMD (DV_0.5_) values within locations at the mid label rate applications (*F* = 0.14; df = 3; *P* = 0.93), significant differences were observed at the maximum label applications between the Front-Alcove (*P* = 0.02) and Alcove-Porch (*P* = 0.02) locations (Table 1). Significant differences in VMD values were also observed between the mid and maximum label rates at the Front (*P* = 0.003) and Porch (*P* = 0.003) locations (Table 1).

**Table 1:** Spray plume droplet density (drops per mm2) and volume median diameter (μm; VMD) of a mosquito adulticide applied within urban Mercer County and suburban Monmouth County, New Jersey, USA. Sampling was conducted within four stations at mid (46.77 ml/ha) and maximum (93.53 ml/ha) label application rates using Florida Latham Bonds (FLB) and Hock rotary impactors and measured by digital analysis.

Location	Impactor type	Application rate	Unit of measure	Sampling station^a^					
				Front	Alcove	Porch	Backyard	Mean	*P*^b^
Urban Mercer	FLB	Mid	Droplet density	110.5 ± 33.4	115.6 ± 20.2	135.8 ± 27.0	135.6 ± 23.7	124.4 ± 12.5	
			(mm^2^ ± SE)						
		Max		136.1 ± 20.9	149.7 ± 30.5	151.1 ± 24.6	162.9 ± 16.3	149.9 ± 11.1	0.06
		Mid	VMD	10.5 ± 0.3	10.9 ± 0.3	10.5 ± 0.3	10.8 ± 0.3	10.7 ± 0.2	
			(DV_0.5_ ± SE)						
		Max		12.8 ± 0.5 a	11.0 ± 1.2 b	12.9 ± 0.3 ac	12.2 ± 0.3 abc	12.2 ± 0.4	0.02
	Hock	Mid	Droplet density	4.4 ± 1.1	5.0 ± 1.1	5.0 ± 0.6	4.8 ± 0.5	4.8 ± 0.4	
			(mm^2^ ± SE)						
		Max		8.7 ± 1.0	6.8 ± 0.8	7.4 ± 1.3	7.4 ± 0.3	7.6 ± 0.5	0.82
		Mid	VMD	13.7 ± 1.9	13.0 ± 1.0	12.2 ± 0.5	14.6 ± 2.3	13.4 ± 0.8	
			(DV_0.5_ ± SE)						
		Max		16.5 ± 0.4	16.7 ± 0.7	16.8 ± 0.8	15.8 ± 0.6	16.4 ± 0.3	0.01
Suburban Monmouth	FLB	Max	Droplet density	131.3 ± 20.6	112.7 ± 22.3	111.6 ± 28.3	101.9 ± 24.6	114.4 ± 11.3	
			(mm^2^ + SE)						
			VMD	14.6 ± 0.9	13.6 ± 0.4	14.2 ± 0.6	13.4 ± 0.7	14.0 ± 0.3	
			(DV_0.5_ + SE)						
	Hock	Max	Droplet density	6.8 ± 1.4	8.0 ± 1.1	6.7 ± 1.2	7.9 ± 0.8	7.3 ± 0.6	
			(mm^2^ + SE)						
			VMD	19.4 ± 1.5	18.4 ± 0.4	18.9 ± 1.0	18.5 ± 1.8	18.8 ± 0.6	
			(DV_0.5_ + SE)						

a All values represented for sampling stations within a row followed by the same letter are not significanlty different (ANOVA, P < 0.05).

b Comparison of combined means for similar units of measure within row (ANOVA, P < 0.05).

*Hock rotating impactors*. During mid label rate applications, we analyzed over 7,800 droplets from all slides, with a mean value of 390.6 drops per station and 195.3 drops per slide (S4 Fig). During maximum label rate applications, we analyzed over 10,000 droplets from all slides, with a mean value of 508.9 drops per station and 254.5 drops per slide (S5 Fig). Aerosolized droplets were collected consistently from all four stations and no significant differences in droplet density were observed by location within the two application rates (*F* = 0.72; df = 3; *P* = 0.55). However, a significant difference in droplet density was observed between the two rates at the Front (*P* = 0.002) and Backyard (*P* = 0.05) locations (Table 1). Additionally, differences in VMD values were observed between the mid and maximum label rates at the Alcove (*P* = 0.03) and Porch (*P* = 0.01) locations (Table 1).

*Differences between FLB and Hock rotating impactors*. Overall, the mean droplet density (± SE) value obtained from FLB impactors in urban Mercer at the mid label rate was 124.37 ± 12.45 mm^2^ and 149.93 ± 11.07 mm^2^ at the maximum label rate, but these values were not significantly different from each other (*F* = 4.70; df = 1; *P* = 0.06) (Table 1). Droplet density obtained from Hock impactors at the mid label application rate was 4.80 ± 0.40 mm^2^ and 7.56 ± 0.45 mm^2^ at the maximum label rate, and again, these values were not significantly different from each other (*F* = 0.06; df = 1; *P* = 0.82) (Table 1). However, droplet density values obtained by the two rotating impactors were significantly different from each other at the mid label (*P* < 0.001) and maximum label (*P* < 0.001) application rates. Additionally, the mean VMD value obtained from FLB impactors at the mid label application rate was 10.68 ± 0.15 μm and 12.24 ± 0.35 μm at the maximum label rate, which were also significantly different from each other (*P* = 0.02). The VMD mean value obtained from Hock impactors at the mid label rate was 13.36 ± 0.76 μm and 16.43 ± 0.3 μm at the maximum label rate, and again, these values were significantly different from each other (*P* < 0.001). The VMD values obtained by the two rotating impactors were also different from each other at the mid label (*P* < 0.001) and maximum label (*P* < 0.001) application rates.

#### Suburban Monmouth County

*FLB and Hock rotating impactors*. Penetration of the spray plume at the maximum application rate was observed on all FLB and Hock rotating impactor slides placed within all stations in suburban Monmouth (S6 and S7 Figs). We analyzed over 21,800 droplets from all FLB slides, with a mean value of 1,284.1 drops per station and 642.1 drops per slide (S6 Fig). We also analyzed over 8,300 droplets from all Hock slides, with a mean value of 490.7 drops per station and 245.3 drops per slide (S7 Fig). Spray droplets were collected from all four stations (Front, Porch, Mid Yard, Backyard) and no significant differences in droplet density were observed between the locations within each impactor type (*F* = 0.23; df = 3; *P* = 0.88) (Table 1). However, droplet density was much larger on FLB impactors and this value significantly differed between the two impactor types at each location (*P* < 0.001). Additionally, no differences were observed in VMD values between the locations within each impactor type (*F* = 0.01; df = 3; *P* = 0.99) (Table 1). However, VMD values were larger for the Hock impactors at each location (*P* < 0.001).

#### Differences between counties by rotating impactor type

**Fig 4:**
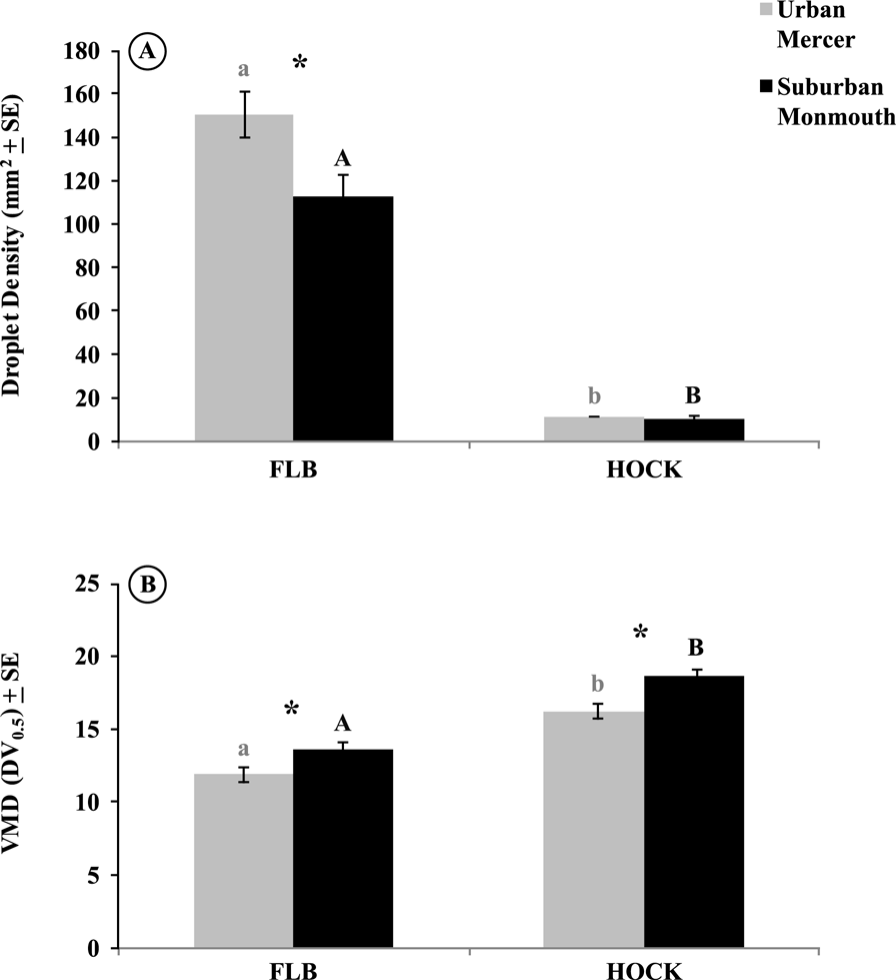
Combined mean values for droplet density (drops per mm^2^) and volume median diameter (μm; VMD) of spray plume from all sampling stations in urban Mercer and suburban Monmouth sampled by both impactor types (FLB and Hock). Treatments with different letters denote significant differences within county by impactor type and asterisks denote significant differences between counties by ANOVA (*P* < 0.05).

Since only maximum label rate applications were conducted in suburban Monmouth, we compared those results with the maximum label applications from urban Mercer. Overall, average droplet density was larger on FLB rotating impactors in urban Mercer (149.93 ± 11.07 mm^2^) than in suburban Monmouth (114.37 ± 11.32 mm^2^), and this difference was found to be significant (*P* < 0.003) (Fig 4A). The mean values for droplet density obtained from Hock impactors was 7.56 ± 0.45 mm^2^ in urban Mercer and 7.28 ± 0.55 mm^2^ in suburban Monmouth; however, no significant differences were found in droplet density gathered by Hock impactors between the counties (*P* = 0.98) (Fig 4A). Additionally, the VMD values obtained from FLB rotating impactors was 12.24 ± 0.35 μm in urban Mercer and 13.95 ± 0.31 μm in suburban Monmouth, which differed significantly from each other (*P* = 0.002) (Fig 4B). Mean VMD values obtained from Hock impactors was 16.43 ± 0.31 μm in urban Mercer and 18.79 ± 0.57 μm in suburban Monmouth, which also differed significantly from each other (*P* < 0.001) (Fig 4B).

### Digital versus manual droplet analysis for Hock impactors

**Fig 5:**
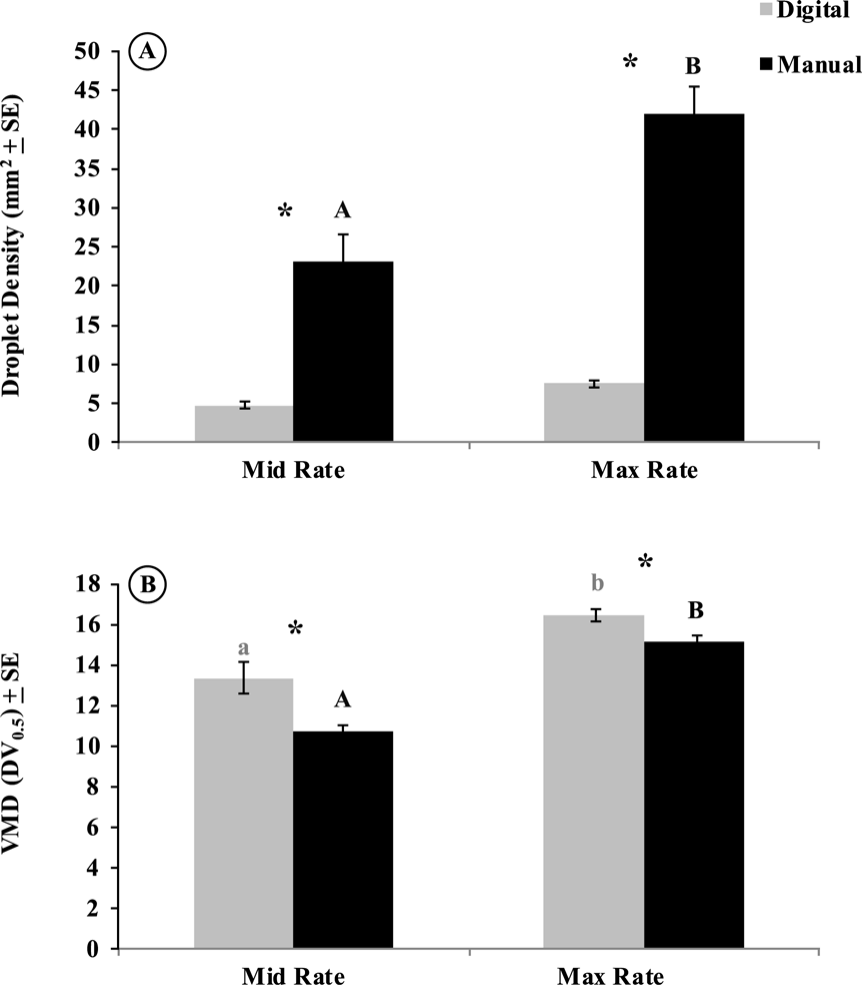
Combined droplet density (drops per mm^2^) and volume median diameter (μm; VMD) of spray plume from all sampling stations in urban Mercer at two different application rates sampled by Hock impactors and recorded by two analysis methods (digital and manual). Treatments with different letters and asterisks denote significant differences by ANOVA (*P* < 0.05).

We compared digital and manual slide reading methods for only Hock impactors in Trenton at mid and maximum rate adulticide applications. At the mid label rate, droplet density was significantly larger (*P* < 0.001) when recorded manually (23.10 ± 3.60 mm^2^) than by the digital technique (4.80 ± 0.40 mm^2^) (Fig 5A). This trend was also consistent at the maximum label rate, with a significantly (*P* < 0.001) larger droplet density recorded by the manual (41.95 ± 3.51 mm^2^) than the digital (7.56 ± 0.45 mm^2^) method (Fig 5A). Additionally, although droplet density was not significantly different between the rates within the digital method (*F* = 0.60; df = 1; *P* = 0.44), a difference was observed within the manual method for the rates (*P* < 0.001) (Fig 5A). We also observed higher VMD values at the mid label rate when comparing the digital (13.36 ± 0.76 μm) and manual (10.74 ± 0.33 μm) methods (*P* < 0.001) (Fig 5B). This pattern was also significant at the maximum label rate for the digital (16.43 ± 0.31 μm) and manual (15.14 ± 0.31 μm) methods (*P* < 0.001) (Fig 5B).

### Meteorological conditions

#### Urban Mercer County

We did not observe thermal inversions before or during the ULV applications at the mid or maximum label rates, which is typical for this highly urbanized environment in northeastern USA.[28] Temperature (19.8 ± 0.1°C) and humidity (84 ± 1.2 RH) were both stable during the mid label and also during the maximum label applications (19.5 ± 0.5°C and 68.5 ± 5.5 RH). Although occasional wind gusts were recorded prior to the experiment, during the ULV applications wind was absent.

#### Suburban Monmouth County

Meteorological data obtained from KNJKEYPO2 indicate that thermal inversions did not occur before or during the ULV applications in Monmouth County, which is also typical for this suburban environment along the Atlantic Coast of northeastern USA. Temperature (14.5 ± 0.8°C) and humidity (90.1 ± 4.2 RH) were both stable during the application and similar to urban Mercer, although occasional wind gusts were recorded leading up to the experiment, during the ULV application, wind was absent.

## Discussion

### Adulticide efficacy on wild mosquito populations and implications for public health

The goal of adulticide applications is the reduction of mosquito populations. Our study did not center on the efficacy of nighttime ULV applications against the diurnally active peridomestic mosquito *Ae*. *albopictus*, but those results have been published beforehand.[10,18,29–31] We have previously shown that nighttime adulticide applications do have an immediate effect on reducing populations of male and female *Ae*. *albopictus* within our experimental sites.[18,30] Although populations rebound quickly after an adulticide application due to the ubiquity of larval habitats such as disposable artificial containers and the continuous broods of emerging adults, we could extend efficacy by conducting a second adulticide application spaced one or two days apart.[10] We determined that dual applications at mid label rates accomplished significantly higher reduction of adults (85.0 ± 5.4%) than single full rate applications (73.0 ± 5.4%).[10] Furthermore, late-season adulticide applications can provide longer relief from biting *Ae*. *albopictus* than earlier applications owing to the lower densities of mosquitoes and their greater vulnerability to adulticides during these cooler periods.[18] However, assessment of insecticide efficacy is highly dependent on appropriate droplet size, density, and penetration in order to offer the greatest probability of killing mosquitoes.

### Droplet size and penetration of ULV aerosols

Droplet size is a crucial factor modulating the efficiency and efficacy of aerosols generated by ULV sprayers[19] because it is directly related to the transport and mortality of the intended mosquito vectors.[32] The most important requirements for an optimal droplet size are that droplets must be small enough to remain airborne, produced in sufficient density for probability of contact with flying mosquitoes, and large enough to impinge readily on the body surface of mosquitoes. The optimum droplet size for mosquito adulticiding is a VMD of 8 to 25 μm.[11,19,20,33] Our field studies consistently collected droplet sizes with a VMD ranging between 10.68 ± 0.15 μm to 18.79 ± 0.57 μm, despite location, rate, or collection method. Additionally, these values were consistent with the pre-calibration VMD (15.18 μm) obtained from a hot-wire calibration instrument. Although differences in VMD were observed between the rates, collection methods, or locations, these differences are not operationally meaningful, as all of our VMD values were consistent with optimum droplet sizes recommended on adulticide labels and previously published reports.[11,19,32]

Droplet penetration of the adulticide into sheltered habitats (such as the alcoves between duplexes or row homes) was one of the primary questions driving these investigations. Because ULV adulticide applications are primarily conducted during the evening or nighttime, *Ae*. *albopictus* may be resting in natural or artificial cryptic habitats, such as alcoves, that are sheltered from the insecticide plume. Few studies have evaluated the movement of aerosols in urban habitats.[34,35] Investigations into the dispersal of adulticides more frequently occur under open field or vegetative canopies, because of the simplicity of these models, and then those theories have been applied to urban habitats.[19,36,37] Our study demonstrated that the aerosol plume from a truck-mounted cold aerosol application penetrates efficiently even into sheltered, cryptic habitats. Our droplet density values were consistent for all locations and no significant differences were observed between locations when using the same application rate or the method of collection. Surprisingly in urban Mercer, both rotating impactor types collected large numbers of droplets even in the alcove location, which was the most sheltered of our sampling stations. Furthermore, since the adulticide was able to penetrate into these sheltered habitats, the novel excitatory component of DUET will flush mosquitoes from resting places and increase their chances of contact with airborne aerosols. The penetration of our urban adulticide application into these habitats has promising potential for vector control programs.

### Droplet density of mid and maximum label rate ULV applications

We found no significant differences using the same collection method between the mean numbers of droplets collected at the two application rates. In contrast, previous authors have reported that to achieve the same efficacy in dense vegetation or urban habitats (versus open field habitats), application rates would have to be increased several fold.[11,38] However, we did not find this to be the case. We achieved the same level of penetration and droplet density at mid label rates as we did at maximum label rate applications. These findings are important for mosquito control programs because newly adopted federal pesticide labels severely limit the amount of active ingredients permissible per acre within a 24 h or annual period. If overall efficacy is not different at mid versus maximum label rates, the lower application rates should be promoted operationally, leading to reduced costs and non-target effects, and greater environmental stewardship. Sophisticated advances in formulations and technology are driving a change in ULV adulticide applications, with the ultimate goal of using the minimum effective volume of the formulated product for maximum efficacy and greater precision and accountability.

### Droplet characteristics within urban and suburban habitats

The penetration of the droplets into the four stations sampled was similar within each county. However, maximum rate applications in urban Mercer displayed a significantly higher droplet density than in suburban Monmouth, as collected on the FLB impactors. This difference may be because of the smaller parcel sizes and shorter spray paths (< 40 m) in urban versus suburban habitats (> 75 m), which would allow a smaller distance between the impactors and the aerosol plume as dispensed by the vehicle, increasing the probability of contact. Previous studies have determined that the most effective spray path is typically 91 to 183 m.[11] The spray paths available in suburban Monmouth are more representative of the habitats in previous investigations, although a single study conducted in urban environments of Thailand using a swath width of 46 m found that dense housing can limit droplet penetration and density.[39] In contrast, our investigations did not find a limiting factor posed by dense urban housing, but rather documented a greater droplet density within urban than in suburban habitats. The extensive network of roadways and alleys available in urban environments actually provide an advantage to truck-mounted adulticide applications by decreasing target distance. This may be an important finding because the greatest threats from mosquito vectors are in urban centers where contact between vectors and hosts are increased.

### Comparison of assay method for droplet collection

Accurate sampling devices are crucial for research associated with measuring size, density, and penetration data of mosquito control aerosols. Any sampling device used for this purpose will exhibit a collection efficiency that is a function of the device itself. However, although a number of methods are available for sampling aerosols, rotary impaction devices are gaining popularity because of their accuracy, efficiency, and ease of use.[25–27,40] Previous studies have found that the FLB impactor collected significantly higher droplet densities as compared to the Hock sampler,[40] and that the FLB impactor always exhibited higher collection efficiencies than the Hock impactor.[25] We also documented differences in droplet density within application rate and county when comparing the two sampling devices. The FLB rotary impactor exhibited a higher droplet density in urban Mercer at mid label, maximum label, and in suburban Monmouth at maximum label. The Hock impactor uses standard 25 mm wide microscope slides and has a low rotational velocity when compared to the FLB impactor which uses 3 mm wide slides and has a 1.5 times higher velocity.[25,27] The smaller surface area of the FLB slides, coupled with their faster velocity, leads to greater collection efficiencies. Our field investigations provide further evidence supporting the use of the FLB rotary impactors, particularly for sampling low-concentrations of ultra-fine aerosols relevant to vector control studies. Repeatability of field-collected data, along with accuracy and reliability of sampling methods are vital in evaluating the efficacy and droplet characteristics of insecticides.

### Meteorological conditions

Meteorology is one of the primary parameters controlling the efficacy and movement of ULV applications. Ultra-low volume adulticides dispensed for mosquito control produce a spray plume composed of ultra-fine droplets that have a low sedimentation velocity and are highly susceptible to atmospheric events.[19] In general, gravity will pull droplets downward and a horizontal wind velocity is required to govern the longitudinal distance that the droplets will travel. Federal pesticide labels instruct that adulticide applications should only be made when wind speed is ≥1.6 km/h and meteorological conditions are favorable for keeping the spray cloud near the ground (e.g., thermal inversion). However, we did not document any thermal inversion and all of our applications were conducted under neutral conditions, a transitory stage where no temperature gradient was recorded. Nevertheless, neutral to weakly stable conditions are considered ideal for ULV spraying[19,28] and the lack of convective motions may have assisted penetration and prevented our adulticide plume from ascending out of the target area. Furthermore, although the lack of wind was also apparent during all of our applications, the reduced droplet density was not as pronounced in urban Mercer as in suburban Monmouth. Although mosquito adulticidal aerosols had penetrated equally into all sampling stations within each county, the lower droplet densities experienced in suburban Monmouth were attributed to the larger parcels and spray paths, which would have been directly influenced by the presence of greater wind speeds. Reduced wind speeds within urban settings, where a close-knit network of roadways and alleys are present, are not as important during nighttime adulticide applications when the nozzle spray velocity of the cold aerosol fogger is able to initiate movement of the droplets within habitats. These findings also hold benefit for mosquito control personnel in domestic environments where the lack of a thermal inversion and reduced wind speeds are normally experienced.

### Comparison of digital versus manual methods of slide readings

The collection of droplets on slides and their subsequent microscopic examination through manual readings by technicians to determine droplet characteristics have been widely used and accepted to assess the quality of adulticide applications.[41] However, manual readings are extremely time consuming and prone to human error, since the technician must randomly select ≥ 200 individual droplets to be measured by conducting visual sweeps across the slide surface. The human eye will naturally navigate towards brighter, larger, or denser areas of the slide. Additionally, droplet density and size determinations must be calculated manually, potentially leading towards additional errors. However, digital methods are gaining popularity because of their speed and accuracy.[29,42] The digital method allows accurate measurement of hundreds of droplets within seconds, with an unbiased determination of VMD and density values. However, little data exists on the comparison of manual and digital methods of droplet density and size determinations. A previous study[25] comparing the digital and manual methods found no measurement differences. Our studies comparing the digital and manual method of slide readings found a significant difference at both application rates for droplet density. In general, droplet density was much lower when determined by digital than by the manual method, and this difference was even more pronounced at the maximum rate applications. This difference could be attributed to the propensity for human readers to gravitate towards more dense areas of the slide, allowing for a quicker reading of a tedious and redundant task. Droplet size (VMD) was also significantly different during both application rates for the digital versus the manual reading methods. Although the VMD values were smaller for the manual method, these numbers were both still within the specifications of federal guidelines and pesticide label recommendations. Because the digital method can quickly measure much larger numbers of droplets and analyze a much more robust dataset, this method may provide a more accurate determination of droplet size and density. As the technology and affordability of these digital systems become more widely available, their routine use by professionals and researchers will lead to more standardized methods of droplet characteristic determinations and more meaningful comparisons between operational and research trials.

Droplet size, density, and penetration are crucial factors modulating the efficacy of aerosol sprays in vector control. Our experiments showed that spray droplets infiltrated all habitats sampled within our field sites, including those most sheltered from the insecticidal cloud. Mid label rates displayed similar droplet density values as maximum rates in urban areas, indicating that lower rates may be used effectively to reduce costs, lessen non-target effects, and increase environmental stewardship. We did not observe a limiting factor posed by dense urban housing, but rather documented a greater droplet density within urban than in suburban habitats. The shorter spray paths, availability of drivable alleys in addition to roads, and the smaller parcel sizes in urban habitats allow for a greater penetration of adulticides into target areas. Our investigations also support the use of the FLB rotary impactors, because of their efficiency in collecting low-concentrations of ultra-fine aerosols relevant to vector control studies. Repeatability of field-collected data, along with accuracy and reliability of sampling methods are vital in evaluating the efficacy and droplet characteristics of insecticides spatially and temporally. We conclude that the digital method of counting and determining droplet dynamics allows for quicker and more accurate measurements, leading to a less biased determination of VMD and density values.

Advances in formulations and technology are driving a change in adulticide applications, leading to use of the minimum effective volume for maximum efficacy and greater precision and accountability. The large and growing populations of *Ae*. *albopictus* in temperate urban centers increase the likelihood of an autochthonous transmission of an arbovirus such as chikungunya or dengue. This may be particularly imminent in the case of chikungunya, as the virus is explosively spreading in the western hemisphere for the first time, having caused over 100,000 human cases in the Caribbean region in only a few months.[7] Absent a human vaccine, we recommend that nighttime applications of ULV adulticides in areas with large populations of *Ae*. *albopictus* be part of an IMM approach for public health protection. Our ultimate objective is to provide vector control operators with appropriate data to base sound judgments when applying adulticides within metropolitan landscapes.

## Supporting Information

S1 Fig Urban alcove stations. Three representative sheltered alcove stations between two adjoining parcels (homes) in urban habitats of sampling sites.
(TIF)

S2 Fig Urban mid label FLB application. Droplet characteristics of a mid label ULV adulticide application within individual stations and parcels in urban Mercer as sampled by FLB type impactors.
(TIF)

S3 Fig Urban max label FLB applications. Droplet characteristics of a maximum label ULV adulticide application within individual stations and parcels in urban Mercer as sampled by FLB type impactors.
(TIF)

S4 Fig Urban mid label Hock application. Droplet characteristics of a mid label ULV adulticide application within individual stations and parcels in urban Mercer as sampled by Hock type impactors.
(TIF)

S5 Fig Urban max label Hock application. Droplet characteristics of a maximum label ULV adulticide application within individual stations and parcels in urban Mercer as sampled by Hock type impactors.
(TIF)

S6 Fig Suburban max label FLB application. Droplet characteristics of a maximum label ULV adulticide application within individual stations and parcels in suburban Monmouth, as sampled by FLB type impactors.
(TIF)

S7 Fig Suburban max label Hock application. Droplet characteristics of a maximum label ULV adulticide application within individual stations and parcels in suburban Monmouth, as sampled by Hock type impactors.
(TIF)
