# Supplementary figures and images for: Droplet Characterization and Penetration of an Ultra-Low Volume Mosquito Adulticide Spray Targeting the Asian Tiger Mosquito, Aedes albopictus, within Urban and Suburban Environments of Northeastern USA
Source: PLoS One. 2016 Apr 26;11(4):e0152069. doi: 10.1371/journal.pone.0152069 (PMC4846071; doi:10.1371/journal.pone.0152069)

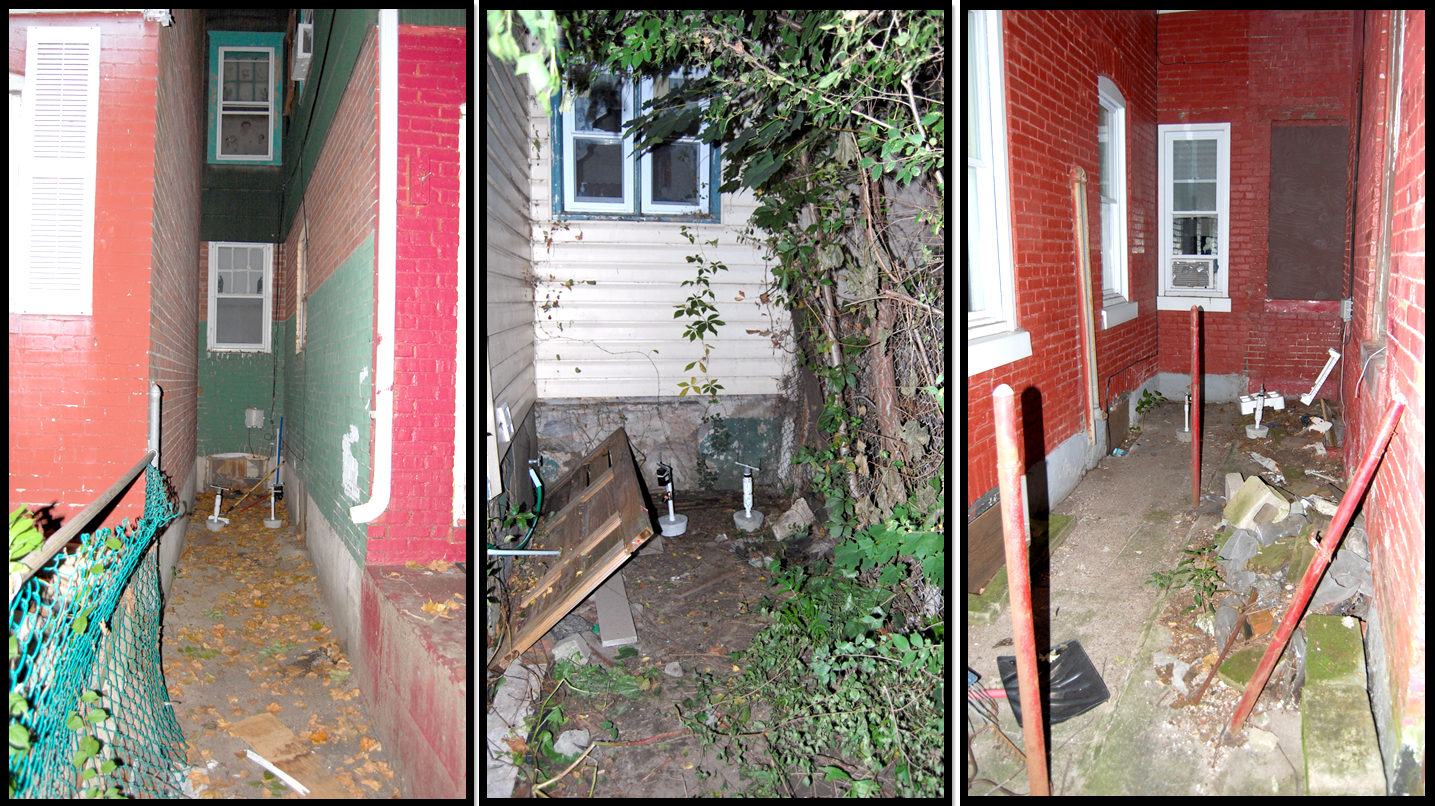

Supplement: S1 Fig — Three representative sheltered alcove stations between two adjoining parcels (homes) in urban habitats of sampling sites. (TIF) [file pone.0152069.s001.tif]

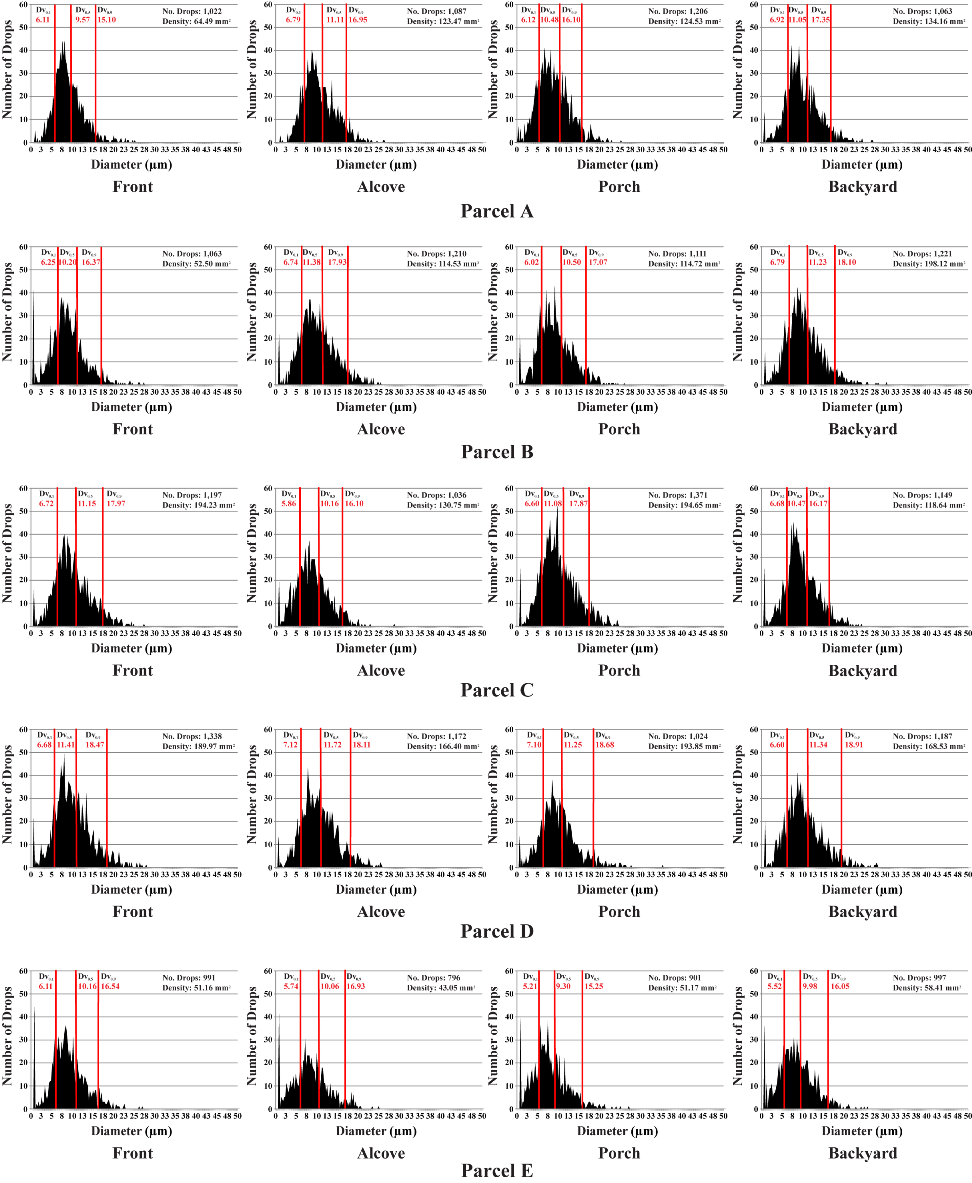

Supplement: S2 Fig — Droplet characteristics of a mid label ULV adulticide application within individual stations and parcels in urban Mercer as sampled by FLB type impactors. (TIF) [file pone.0152069.s002.tif]

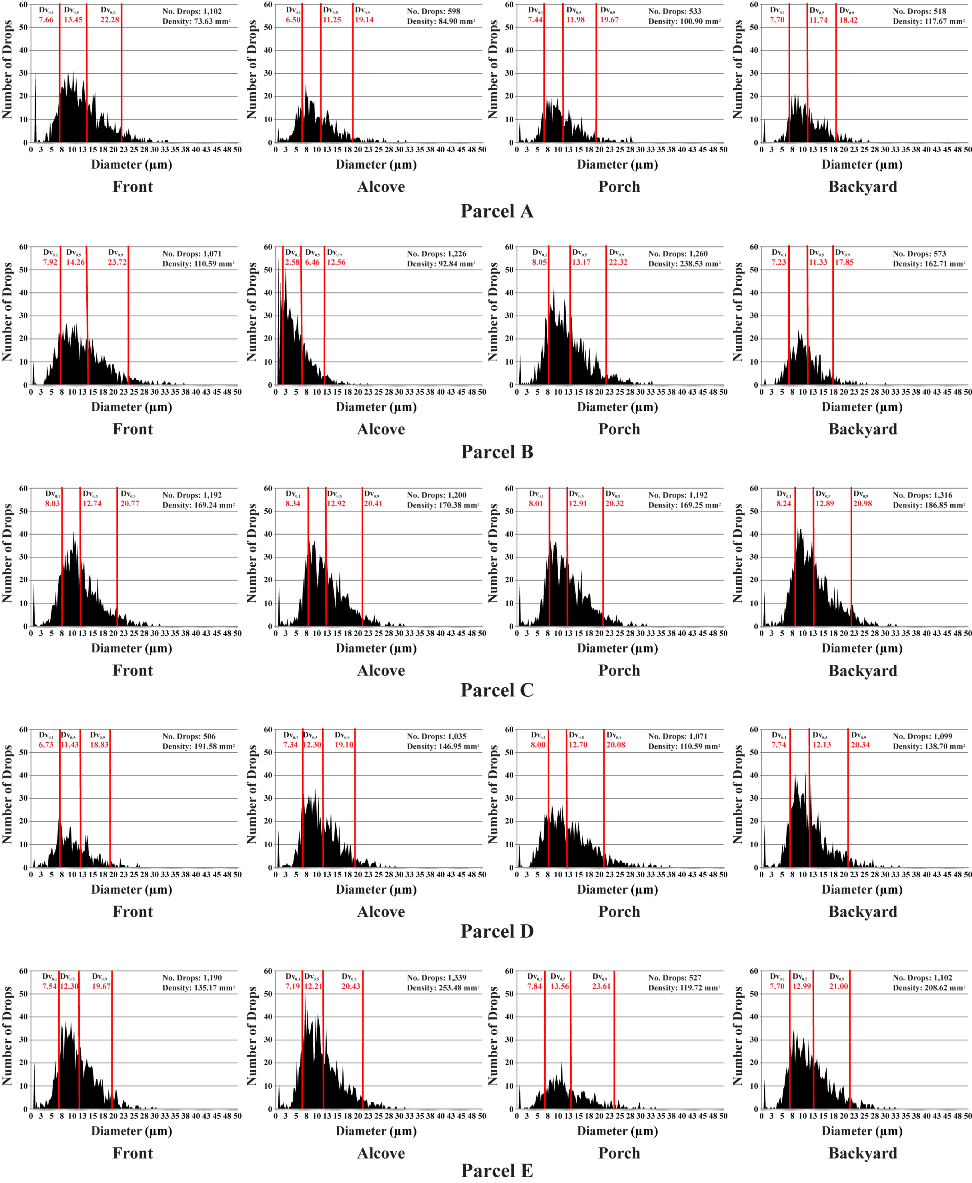

Supplement: S3 Fig — Droplet characteristics of a maximum label ULV adulticide application within individual stations and parcels in urban Mercer as sampled by FLB type impactors. (TIF) [file pone.0152069.s003.tif]

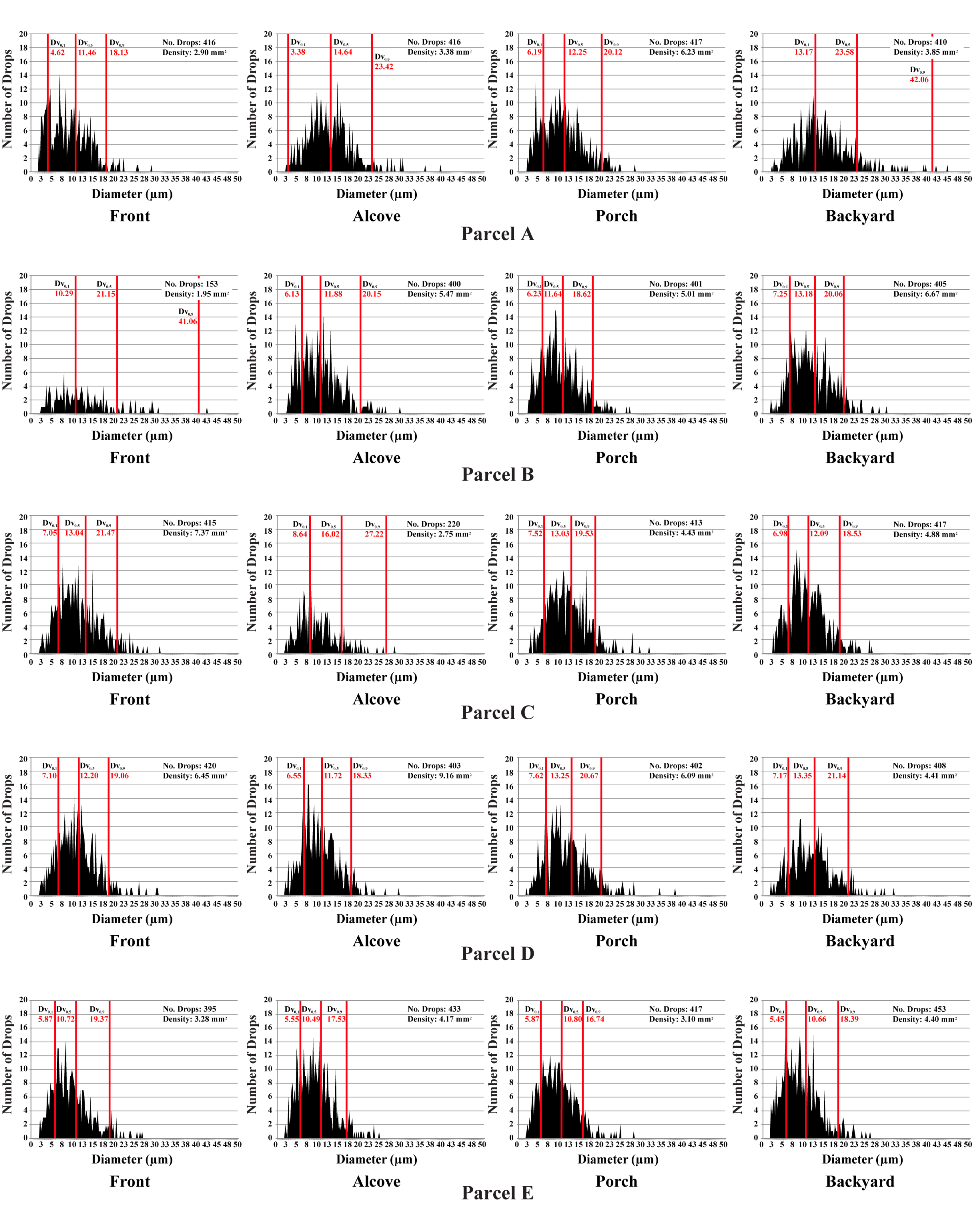

Supplement: S4 Fig — Droplet characteristics of a mid label ULV adulticide application within individual stations and parcels in urban Mercer as sampled by Hock type impactors. (TIF) [file pone.0152069.s004.tif]

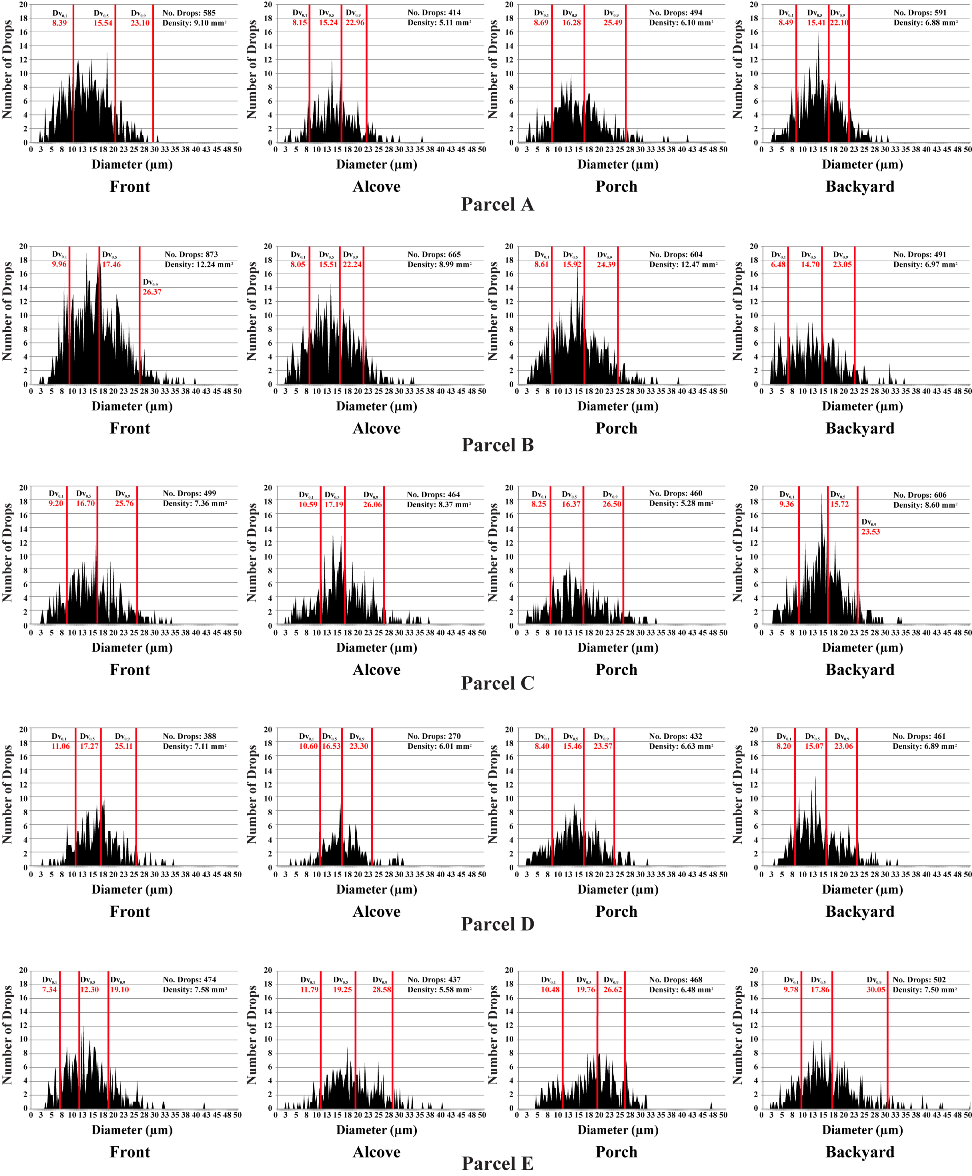

Supplement: S5 Fig — Droplet characteristics of a maximum label ULV adulticide application within individual stations and parcels in urban Mercer as sampled by Hock type impactors. (TIF) [file pone.0152069.s005.tif]

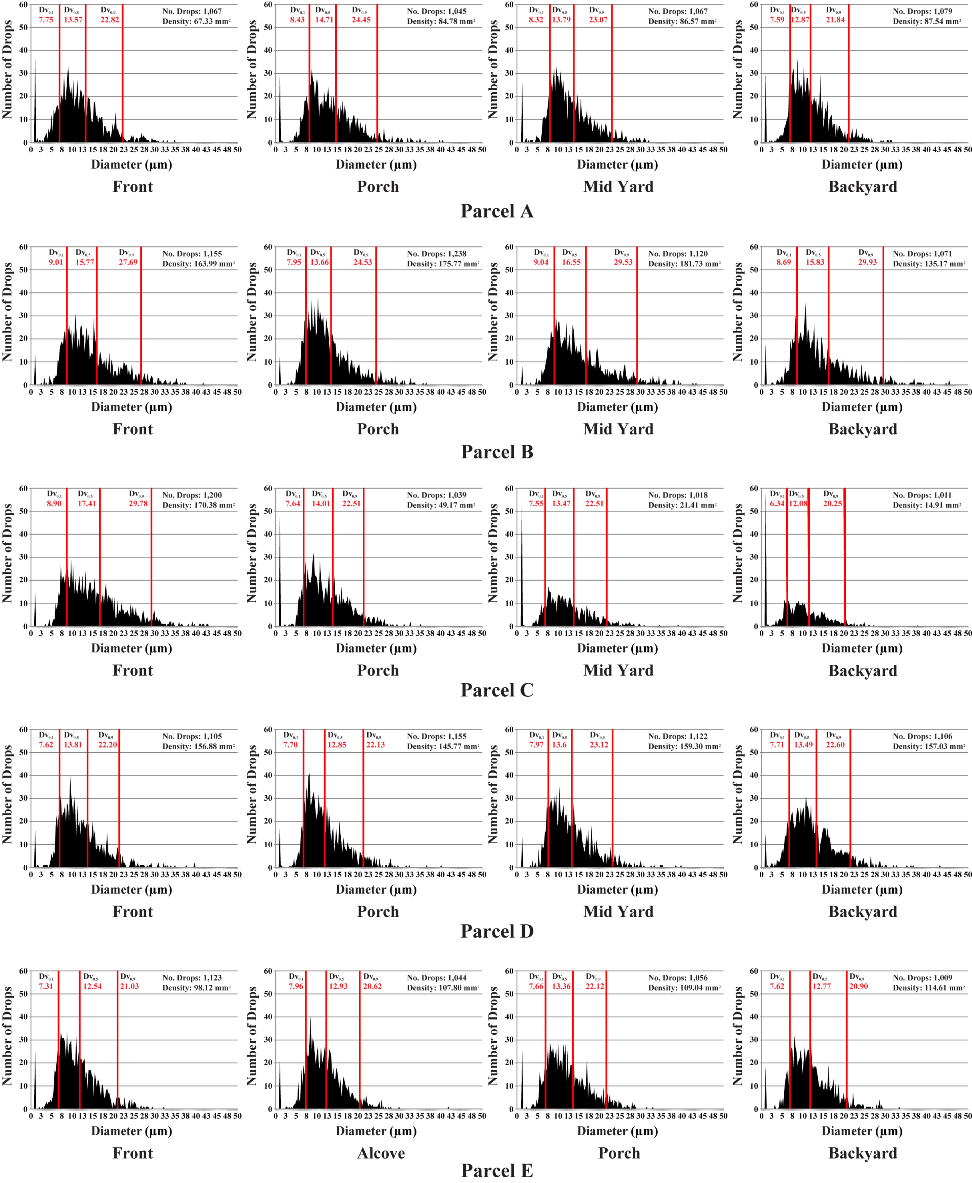

Supplement: S6 Fig — Droplet characteristics of a maximum label ULV adulticide application within individual stations and parcels in suburban Monmouth, as sampled by FLB type impactors. (TIF) [file pone.0152069.s006.tif]

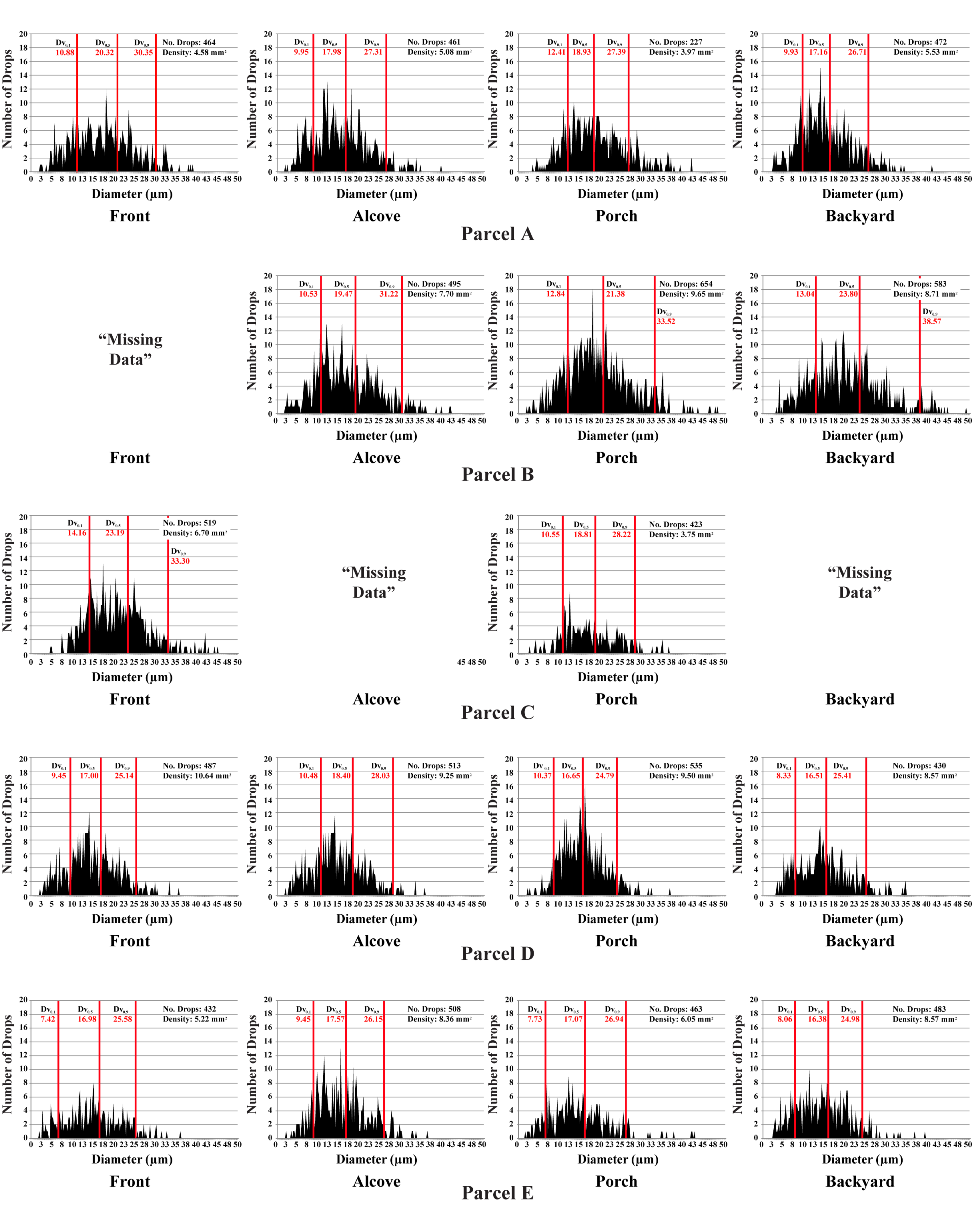

Supplement: S7 Fig — Droplet characteristics of a maximum label ULV adulticide application within individual stations and parcels in suburban Monmouth, as sampled by Hock type impactors. (TIF) [file pone.0152069.s007.tif]
